# A Fuzzy Tuned and Second Estimator of the Optimal Quaternion Complementary Filter for Human Motion Measurement with Inertial and Magnetic Sensors

**DOI:** 10.3390/s18103517

**Published:** 2018-10-18

**Authors:** Xiaoyue Zhang, Wan Xiao

**Affiliations:** School of Instrumentation and Optoelectronic Engineering, Beihang University, Beijing 100191, China; xw7586@buaa.edu.cn

**Keywords:** human motion measurement, sensor fusion, complementary filter, fuzzy logic, inertial and magnetic sensors, ESOQ-2

## Abstract

To accurately measure human motion at high-speed, we proposed a simple structure complementary filter, named the Fuzzy Tuned and Second EStimator of the Optimal Quaternion Complementary Filter (FTECF). The FTECF is applicable to inertial and magnetic sensors, which include tri-axis gyroscopes, tri-axis accelerometers, and tri-axis magnetometers. More specifically, the proposed method incorporates three parts, the input quaternion, the reference quaternion, and the fuzzy logic algorithm. At first, the input quaternion was calculated with gyroscopes. Then, the reference quaternion was calculated by applying the Second EStimator of the Optimal Quaternion (ESOQ-2) algorithm on accelerometers and magnetometers. In addition, we added compensation for accelerometers in the ESOQ-2 algorithm so as to eliminate the effects of limb motion acceleration in high-speed human motion measurements. Finally, the fuzzy logic was utilized to calculate the fusion factor for a complementary filter, so as to adaptively fuse the input quaternion with the reference quaternion. Additionally, the overall algorithm design is more simplified than traditional methods. Confirmed by the experiments, using a commercial inertial and magnetic sensors unit and an optical motion capture system, the efficiency of the proposed method was more improved than two well-known methods. The root mean square error (RMSE) of the FTECF was less than 2.2° and the maximum error was less than 5.4°.

## 1. Introduction

Human motion measurement is a key technology in rehabilitation, gait analysis, man–machine interaction, virtual reality, and other fields [1,2,3,4]. There are numerous kinds of human motion measurement techniques such as mechanical, magnetic, optical, acoustic, and inertial/magnetic. Most of these techniques require emissions from a source so as to track objects [5]. The magnetic measurement technique requires a self-excited stable magnetic field. The ultrasonic measurement technique needs to transmit ultrasonic waves. The optical measurement technique requires light to illuminate objects. However, no self-emission source is required for the inertial/magnetic measurement technique.

Motion measurement using inertial and magnetic sensors is a relatively new technique, which has received wide attention in recent years [4]. The inertial/magnetic measurement technique often uses a combination of micro-electromechanical system (MEMS) gyroscopes, accelerometers, and magnetometers, called magnetic, angular rate, and gravity (MARG) sensors [6]. MEMS sensors are usually low-cost, small in size, and can be manufactured into a wrist watch size [4], which is suitablefor the data collection of wearable devices. There are two advantages in the inertial/magnetic measurement technique, namely: one, is that the measurement technique itself has no inherent latency, and all delays are attributed to the data transmission, which is conducive to real-time measurement; and the other, is its lack of a necessary self-emission source [5]. This makes inertial/magnetic measurement systems easy to carry and use; moreover, the scope of use is not limited to a certain area.

We can obtain the motion of a human body by measuring the posture of the main limbs. In general, human motion can be seen as the movement of a kinematic chain composed of multiple independent limbs, and the members are bound by the connections between them. After the MARG sensors module has been fixed onto the main limbs of a human, the posture of the entire human body can be obtained by measuring the posture of each limb relative to the reference coordinate frame fixed with the earth [4]. According to the different data characteristics of the gyroscope, accelerometer, and magnetometer, a corresponding data fusion method is needed.

There are two principal methods of data fusion: the Kalman filter algorithm and the complementary filtering algorithm. The Kalman filter algorithm focuses on how to solve the effects of linear acceleration, environmental magnetic field abruption [7], and accelerometer and magnetometer data preprocessing algorithms [4]. There are also variations of the Kalman filter algorithm such as extended Kalman filtering [7,8] and the particle Kalman filter algorithm [9]. Time delay is one of the main downsides of the Kalman filter algorithm methods. The complementary filter algorithm is a method of data fusion for the MARG sensors in the frequency domain. The complementary filter algorithm mainly focuses on how to mix the accelerometer and magnetometer data so as to generate corrections, which can modify the quaternion calculated with the gyroscope data [5,10]. In addition, there is the gradient descent method that calculates attitudes by using an analytically derived and optimized gradient descent algorithm [11].

The purpose of this paper was to propose a simple structure complementary filtering algorithm that was suitable for measuring high dynamic human motion. In the current research, the complementary filter algorithm usually adopts a fixed conversion frequency, which is often difficult to adapt to high dynamic human motion. To address this problem, we proposed a complementary filter algorithm based on fuzzy logic and the Second EStimator of the Optimal Quaternion (ESOQ-2) algorithm. The fuzzy tuned algorithm was used to adjust the conversion frequency adaptively, which improved the adaptability of the algorithm for high dynamic human motion. The compensation of the accelerator for the ESOQ-2 algorithm can improve the adaptability of the proposed algorithm for high dynamic human motion. The MARG sensors’ data were then input into the Fuzzy Tuned and Second EStimator of the Optimal Quaternion Complementary Filter (FTECF) to calculate the body posture. Then, the result was compared with the optical reference attitudes. The experimental results verified the performance of the proposed algorithm.

In Section 2, the basic definitions and details of the proposed algorithm are provided. Section 3 explains the measurement experiments with MARG sensors, and is devoted to the interpretation of the results. The final section provides some conclusions and future work.

## 2. Materials and Methods

This section is divided into two parts, the material used in the proposed algorithm (Section 2.1, Section 2.2 and Section 2.3) and the details of the proposed algorithm (Section 2.4, Section 2.5 and Section 2.6).

### 2.1. Coordinate System Definition

**Figure 1:**
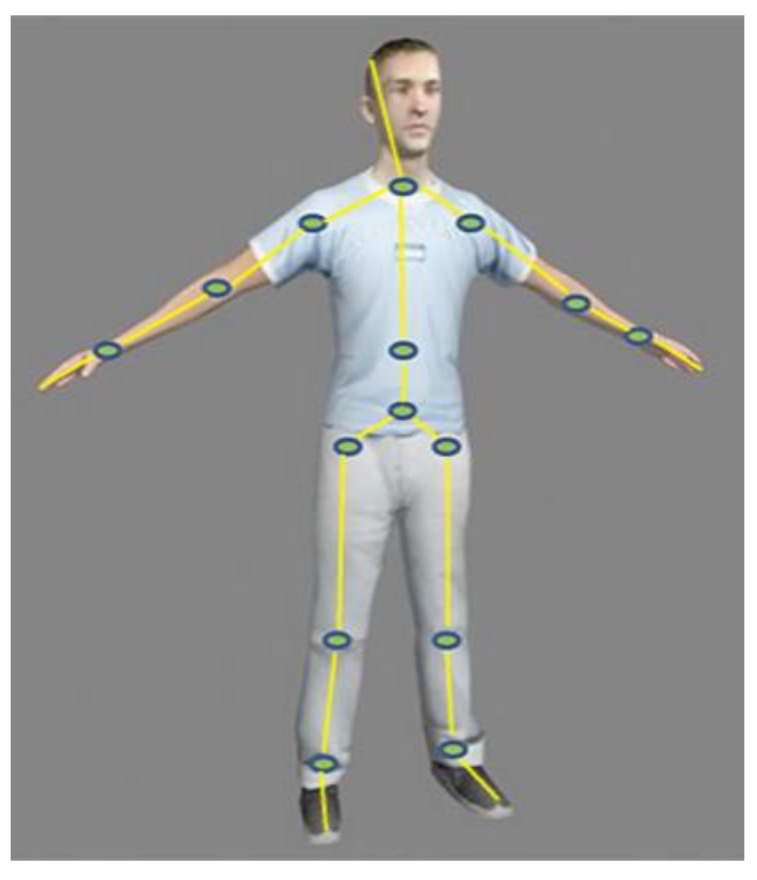
The segmentations of human limbs.

The inertial/magnetic measurement technique often refers to the body’s limbs as rigid bodies [3]. The human body can be represented by 15 to 19 rigid body models that are connected to each other [12,13], so the overall body motion can be obtained by measuring the posture of each limb [9]. The human limbs are shown in Figure 1.

In this paper, we studied the generic limb posture measurement, which can be applied to all major limbs of the body. The upper limb movement is more agile and flexible than the other body limbs [9]. By convention, we chose the upper limb as the main research object [3,4,6,9,10]. In the upper limb, the more flexible upper arm movement was selected for detailed study.

**Figure 2:**
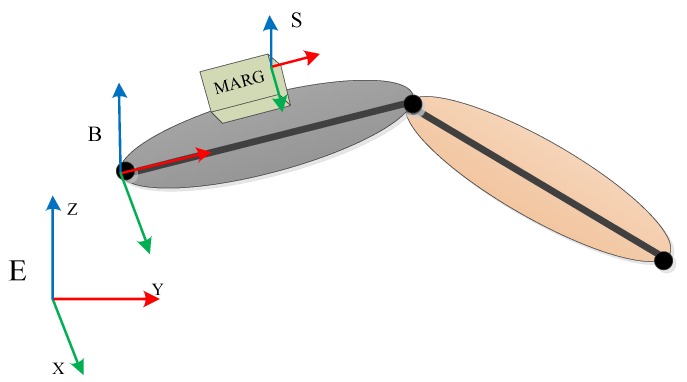
The definitions of the coordinate frames. E is the reference coordinate frame, which is mounted on the earth; B is the coordinate frame of the upper arm; S is the coordinate frame of the magnetic, angular rate, and gravity (MARG) sensor.

The definition of the coordinate frames involved in the upper arm is illustrated in Figure 2. For the upper arm and MARG sensors, the coordinate frame was defined as B and S, respectively. In addition, the reference coordinate frame mounted on the earth was also defined as E.

The reference coordinate for frame E is defined according to the orientation of the human body at the beginning of the measurement. The origin of the reference coordinate frame is in the vicinity of the human body in space. The directions of the three axes are defined as the Z-axis (upward in the direction of the gravity vector), the X-axis (pointing to the right of the body), and the Y-axis (pointing to the front of the body), which follow the right-handed coordinate system. Once the reference coordinate frame is defined, it is fixed in the space and does not change with the movement of the human body.

The coordinate of frame B is fixed to the human skeleton, and the origin can be set along the skeleton, usually at the rotation center of the limb. The T-pose is where the arms are straight forward, with the palms facing down and the thumb pointing straight ahead [14]. The upper arm coordinate frame and the reference coordinate frame maintain the same direction at the T-pose, and can be transformed into each other through a translation in space [10].

The origin of the coordinate of frame S is at the center of the three axes of the accelerometer, axially along the housing of the MARG sensors, and the directions of the three axes follow the right-handed coordinate system. The data output of each sensor is represented in the corresponding sensor coordinate frame.

For the sake of simplicity, we assumed that there was no relative displacement between the sensor and the upper arm, so the sensor coordinate frame and upper arm coordinate frame were considered to be identical. That is, the two frames had the same orientation, but with a different displacement.

### 2.2. Representation

The upper arm’s movement information can be represented with Euler angles or quaternions [3]. This paper used the quaternion to calculate the rotational movement of the limb, and converted the quaternion into Euler angles for visual representation. The advantage of the Euler angles representation method is that it can intuitively represent a rotation. However, the Euler angles representation method is prone to the gimbal lock problem, resulting in the appearance of singularities. In contrast, the quaternion representation method can avoid the occurrence of singularities, and is more computationally efficient. In addition, the quaternion can be transformed with the attitude matrix and Euler angles [15].

In previous studies [4,5,6], the motion of the upper arm was usually described by the kinematics differential (Equation (1)).

(1) $${\dot{q}}_{B}^{E}=\frac{1}{2}q_{B}^{E}⊗{\bar{\omega }}_{B}$$

where $q_{B}^{E}$ denotes the quaternion from the upper arm coordinate frame, *B*, to the reference coordinate frame, *E*, which is calculated based on the gyroscope measurement. ${\bar{\omega }}_{B}=\left[\begin{array}{cc} 0 & \omega_{B}^{T} \end{array}\right]$ represents a four-element column vector, and $\omega_{B}=\left[\begin{array}{ccc} \omega_{B,x} & \omega_{B,y} & \omega_{B,z} \end{array}\right]$ is the measured value of the triaxial gyro in the upper arm coordinate frame.

We can express a quaternion as follows:

(2) $$q=q_{0}+q_{vert}=q_{0}+q_{1}i+q_{2}j+q_{3}k$$

Then, Equation (1) can be written as a matrix as follows:

(3) $$\left[\begin{array}{c} {\dot{q}}_{B,0}^{E}\\ {\dot{q}}_{B,1}^{E}\\ {\dot{q}}_{B,2}^{E}\\ {\dot{q}}_{B,3}^{E} \end{array}\right]=\frac{1}{2}\left[\begin{array}{cccc} 0 & -\omega_{B,x} & -\omega_{B,y} & -\omega_{B,z}\\ \omega_{B,x} & 0 & \omega_{B,z} & -\omega_{B,y}\\ \omega_{B,y} & -\omega_{B,z} & 0 & \omega_{B,x}\\ \omega_{B,z} & \omega_{B,y} & -\omega_{B,x} & 0 \end{array}\right]\left[\begin{array}{c} q_{B,0}^{E}\\ q_{B,1}^{E}\\ q_{B,2}^{E}\\ q_{B,3}^{E} \end{array}\right]$$

If the gyroscope output, ${\bar{\omega }}_{B,t}$, and the fused quaternion, $q_{t-\Delta t}^{E}$, are known, then we can obtain the input quaternion as follows:

(4) $$q_{gyr,t}^{E}=q_{t-\Delta t}^{E}+{\dot{q}}_{gyr,t}^{E}\Delta t$$

where Δt is the sampling time.

**Figure 3:**
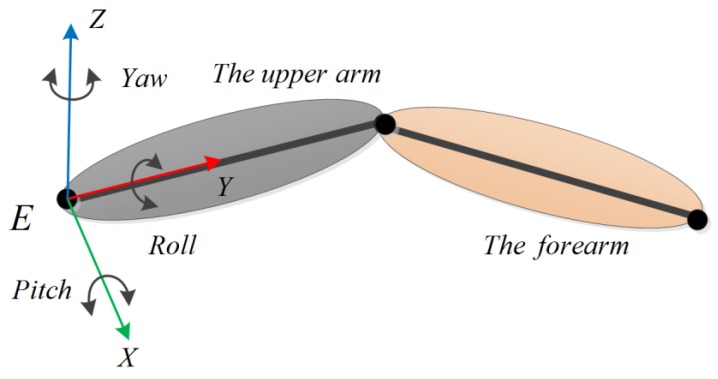
The definitions of the Euler angles.

The motion of the upper arm can be represented by Euler angles (pitch–roll–yaw), as in Figure 3. The pitch angle is a rotation angle with respect to the X-axis of the coordinate frame, *E*. Similarly, we can define the roll angle and the yaw angle.

### 2.3. Motion Speed

The maximum movement frequency of the upper arm of the human body is about 3.7 times per second [16], therefore, it is difficult for a person to maintain long-term high-speed movements. In this study, we choose two representative human motion speeds, namely: 0.5 movements per second (0.5 mov/s) and 2 movements per second (2 mov/s), representing human low-speed motion and human high-speed motion, respectively.

### 2.4. Description of the Proposed Algorithm

**Figure 4:**
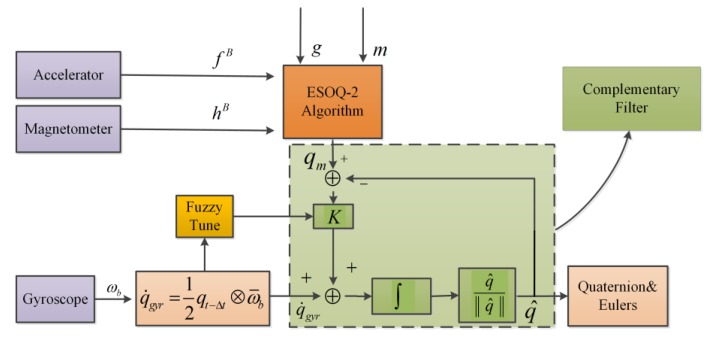
Block diagram of the proposed algorithm.

We proposed a complementary filter algorithm based on fuzzy logic and the ESOQ-2 algorithm. The block diagram of the algorithm is shown in Figure 4. The ESOQ-2 algorithm calculates the reference quaternion with accelerometers and magnetometers. The reference quaternion has a more precise dynamic response at a low frequency. In contrast, the input quaternion calculated by gyroscopes has a more precise dynamic response in high frequency. In this paper, a complementary filter fused the reference quaternion and input quaternion, and the conversion frequency was tuned by fuzzy logic.

**Figure 5:**
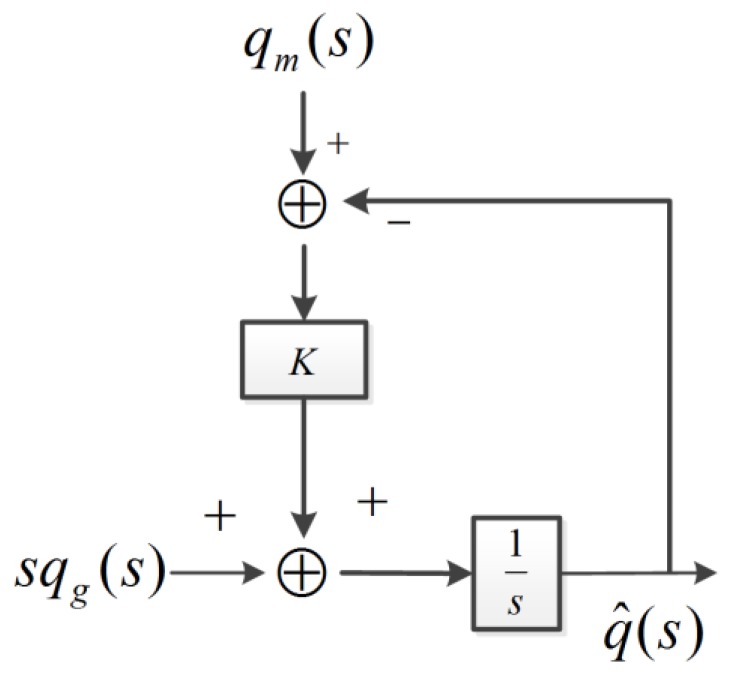
The frequency domain representation of the complementary filter.

In order to analyze the complementary filter, we performed a Laplace transform (*s* is the Laplace operator) on $q_{m}$, ${\dot{q}}_{gyr}$, and $\hat{q}$. As shown in Figure 5, $q_{m}(s)$ is the Laplace transformation (LT) of $q_{m}$, and $sq_{gry}(s)$ denotes the LT of ${\dot{q}}_{gyr}$. $F_{1}(s)$ and $F_{2}(s)$ are two transfer functions and $F_{1}(s)+F_{2}(s)=1$ [5].

(5) $$F_{1}(s)=\frac{\hat{q}(s)}{q_{m}(s)}=\frac{K}{s+K}=\frac{1}{\tau s+1}\;$$

(6) $$F_{2}(s)=\frac{\hat{q}(s)}{q_{gry}(s)}=\frac{s}{s+K}=\frac{\tau s}{\tau s+1}\;$$

(7) $$\hat{q}\left(s\right)=F_{1}(s)q_{m}(s)+F_{2}(s)q_{gry}(s)=\frac{K}{s+K}q_{m}(s)+\frac{s}{s+K}q_{gry}(s)\;$$

(8) $$f_{c}=\frac{1}{2\pi \tau }=\frac{K}{2\pi }\;$$

where $\tau =\frac{1}{K}$.

In Equation (7), $F_{1}(s)$ is equivalent to a low-pass filter and can filter out the high-frequency noise of the reference quaternion. $F_{2}(s)$ is equivalent to a high-pass filter, and can filter out the low frequency noise of the input quaternion. The conversion frequency of the complementary filter is $f_{c}$, in Equation (8), and can be adjusted by varying the filter gain, *K* [17].

The output of the complementary filter in the time domain in Figure 4 is as follows:

(9) $${\hat{q}}_{t}=[K(q_{m}-{\hat{q}}_{t})+{\dot{q}}_{gyr}]\Delta t+q_{t-\Delta t}\;$$

By applying Equation (4) to Equation (9), we get the following [6]:

(10) $${\hat{q}}_{t}=(1-\frac{K\Delta t}{1+K\Delta t})q_{gry,t}^{E}+\frac{K\Delta t}{1+K\Delta t}q_{m,t}^{E}\;$$

(11) $${\hat{q}}_{t}=\;(1-\mu_{t})q_{gry,t}^{E}+\mu_{t}q_{m,t}^{E},0\leq \mu_{t}\leq 1.\;$$

where $\mu_{t}=\frac{K\Delta t}{1+K\Delta t}=1-\frac{1}{1+K\Delta t}=1-\frac{1}{1+2\pi f_{c}\Delta t}$, by using Equation (8) and $f_{c}\propto \mu_{t}$. Therefore, it can be seen from Equation (11) that tuning the fusion factor, $\mu_{t}$, can change the conversion frequency, $f_{c}$, of the complementary filter. When $\mu_{t}$ increases, $f_{c}$ correspondingly increases. At this time, the complementary filter output is more biased toward the reference quaternion, $q_{m,t}^{E}$. Similarly, when $\mu_{t}$ decreases, $f_{c}$ decreases correspondingly, and the complementary filter is more biased to the input quaternion, $q_{gry,t}^{E}$.

To prevent the quaternion from being non-orthogonal, we performed orthogonalization on it.

(12) $$\hat{q}=\frac{1}{\sqrt{{\hat{q}}_{0}^{2}+{\hat{q}}_{1}^{2}+{\hat{q}}_{2}^{2}+{\hat{q}}_{3}^{2}}}\left[\begin{array}{c} {\hat{q}}_{0}\\ {\hat{q}}_{1}\\ {\hat{q}}_{2}\\ {\hat{q}}_{3} \end{array}\right]$$

### 2.5. The ESOQ-2 Algorithm and Computation for the Accelerator

#### 2.5.1. The ESOQ-2 Algorithm and Reference Quaternion

We calculated the reference quaternion by using the ESOQ-2 algorithm. The ESOQ-2 algorithm has been proposed for the Wahba problem [18]. The Wahba problem is used to estimate the attitudes of a body in the least-squares sense, by using the vector’s reference values and the corresponding measurement values, as shown in Equation (13).

(13) $$L_{W}(A)=\frac{1}{2}\sum_{i-1}^{n}\alpha_{i}{\left\|Ar_{i}-b_{i}\right\|}^{2}=\min$$

where $\alpha_{i}$ represents the relative weight of the observation vector ($\sum_{i}\alpha_{i}=1,i=1\sim n$). The weight is related to the data credibility of the *i*th observation vector.

In Equation (13), *r* is the *n*-dimensional vector defined in the reference coordinate frame, *b* is the corresponding vector definition in the body coordinate, and *A* is the attitude matrix from *r* to *b*. In this paper, *n* is 2, *r* represents the gravity vector and geomagnetic field vector defined in the reference coordinate frame, and *b* represents the accelerometer and magnetometer measurement vector. Assuming that in the quasi-static condition (limb motion acceleration is negligible when compared to gravitational acceleration) the magnetic field is not distorted, the attitude measurement in this paper was used to determine the body posture through the reference and measurement values of the gravity vector and the geomagnetic field vector.

The *q*-method demonstrates that the optimal quaternion, $q_{opt}$, is the eigenvector with the largest eigenvalue of the 4×4 symmetric matrix *K* [19],

(14) $$Kq_{opt}=\lambda_{max}q_{opt}$$

Therefore, as long as the eigenvector with the largest eigenvalue of matrix *K* is obtained, the optimal attitude quaternion can be obtained.

The procedure for the ESOQ-2 algorithm is as follows:

(1) Calculate the structure matrix

(15) $$K=\left[\begin{array}{cc} B+B^{T}-tr[B]I_{3\times 3} & z\\ z^{T} & tr[B] \end{array}\right]$$

where $B=\sum_{i}\alpha_{i}b_{i}r_{i}^{T}$ is the attitude profile matrix, $I_{3\times 3}$ is the 3×3 unit matrix, and *z* is the vector $z=\sum_{i}\alpha_{i}b_{i}\times r_{i}={\left\{B(2,3)-B(3,2),B(3,1)-B(1,3),B(1,2)-B(2,1)\right\}}^{T}$.

(2) Calculate the maximum eigenvalue of the approximated matrix *K*

(16) $$\lambda_{\max}=\frac{1}{2}(\sqrt{2\sqrt{d}-b}+\sqrt{-2\sqrt{d}-b})$$

where $b=-2(tr[B])+tr[adj(B+B^{T})]-z^{T}z$, d=det(K).

(3) Calculate the reference quaternion

From the formula $[(tr[B]-\lambda_{\max})S-zz^{T}]e=Me=0$, the best robustness vector can be obtained, so

(17) $$q=\left\{\begin{array}{c} (\lambda_{\max}-tr[B])e_{k}\\ z^{T}e_{k} \end{array}\right\}$$

Thus, the direction of the optimal quaternion is obtained by normalizing *q*, that is, $q_{opt}=q/\sqrt{q^{T}q}$.

(4) Avoid singularity adjustment

For the singularity problem that this method may produce, the specific details of the solution are in the literature [19].

In this paper, the gravity vector is expressed as $g={\left[\begin{array}{ccc} 0 & 0 & 9.78 \end{array}\right]}^{T}$, assuming that the local geomagnetic vector modulus is *h*, and the declination angle of the local geomagnetic vector is ε. When the X-axis of the reference coordinate frame points to the east and the Y-axis points to the north, the geomagnetic vector can be expressed as $m={\left[\begin{array}{ccc} 0 & h\cos\varepsilon & h\sin\varepsilon \end{array}\right]}^{T}$.

#### 2.5.2. Compensation for the Accelerator

The quasi-static condition in the ESOQ-2 algorithm is rare in human motion. Therefore, we needed to compensate the accelerator in order to handle a high dynamic movement. Because of the acceleration of the limb movement, the accelerometer output is the combination of the gravity acceleration and the motion acceleration. In this case, the input quaternion is more reliable, so we replaced the accelerometer’s output with the gravitational acceleration vector calculated with the input quaternion. Based on the aforementioned concept, the input vectors, *f*, (gravity-related) for the ESOQ-2 algorithm were calculated using the following equations:

(18) $$f^{B}=\{\begin{array}{c} \frac{f_{m,t}}{\left\|f_{m,t}\right\|},if\left|\left\|f_{m,t}\right\|-\left\|g\right\|\right|\leq \delta_{a}\;and\;\left\|\omega_{b,t}\right\|\leq \delta_{w}，\\ C_{E}^{B}\frac{g}{\left\|g\right\|},otherwise. \end{array}$$

(19) $$\left\{ \begin{array}{l} \left\|f_{m,t}\right\|=\sqrt{f_{mx,t}^{2}+f_{my,t}^{2}+f_{mz,t}^{2}}\\ \left\|\omega_{b,t}\right\|=\sqrt{\omega_{bx,t}^{2}+\omega_{by,t}^{2}+\omega_{bz,t}^{2}} \end{array}\right.$$

(20) $$C_{E}^{B}=\left[\begin{array}{ccc} q_{gyr,0}^{2}+q_{gyr,1}^{2}-q_{gyr,2}^{2}-q_{gyr,3}^{2} & 2\left(q_{gyr,1}q_{gyr,2}+q_{gyr,0}q_{gyr,3}\right) & 2\left(q_{gyr,1}q_{gyr,3}-q_{gyr,0}q_{gyr,2}\right)\\ 2\left(q_{gyr,1}q_{gyr,2}-q_{gyr,0}q_{gyr,3}\right) & q_{gyr,0}^{2}-q_{gyr,1}^{2}+q_{gyr,2}^{2}-q_{gyr,3}^{2} & 2\left(q_{gyr,2}q_{gyr,3}+q_{gyr,0}q_{gyr,1}\right)\\ 2\left(q_{gyr,1}q_{gyr,3}+q_{gyr,0}q_{gyr,2}\right) & 2\left(q_{gyr,2}q_{gyr,3}-q_{gyr,0}q_{gyr,1}\right) & q_{gyr,0}^{2}-q_{gyr,1}^{2}-q_{gyr,2}^{2}+q_{gyr,3}^{2} \end{array}\right]$$

where $f_{m,t}=\left[\begin{array}{ccc} f_{mx,t} & f_{mx,t} & f_{mx,t} \end{array}\right]$ is the accelerometer triaxial output data, and $\omega_{b,t}$ is the gyroscope triaxial output data. $C_{E}^{B}$ represents the attitude transformation matrix from the reference coordinate frame (E) to the sensor coordinate frame (*S*) and the upper arm coordinate frame (*B*), assuming that the latter two coordinate frames are consistent in direction. $q_{gyr}=\left[\begin{array}{cccc} q_{gyr,0} & q_{gyr,1} & q_{gyr,2} & q_{gyr,3} \end{array}\right]$ is the input quaternion. $\delta_{a}$ and $\delta_{w}$ are the corresponding thresholds for acceleration and angular velocity, respectively.

### 2.6. Quaternion Fusion Factor

In this paper, fuzzy logic was used to generate the fusion factor for the reference quaternion and the input quaternion. As a fuzzy input, *e*1 can be calculated as Equation (21), as follows:

(21) $$e1=\frac{\xi }{\xi +\left\|{\dot{q}}_{gyr}^{E}\right\|}$$

where the quaternion differential value, ${\dot{q}}_{gyr}$, is calculated from the gyroscope output. In addition, ξ represents the minimum value of the angular velocity differential mode value $\left\|{\dot{q}}_{gyr}^{E}\right\|$ under normal human motion, which can be obtained through simple experiments.

The specific steps for designing a fuzzy controller are as follows [20]:

(1) Fuzzification

That is, a fuzzy set is used to represent real-valued signals. This paper used a single-valued method.

(2) Establish fuzzy inference rules

In this part, *e*1 is the fuzzy input, and u is the fuzzy output. The fuzzy output, u, was $\mu_{t}$ in the paper. From Equation (21), we can see that when the acceleration of the line motion was large, ${\dot{q}}_{gyr}$ increased and *e*1 decreased. According to Equation (11), $\mu_{t}$ should be tuned to be smaller, as the quaternion calculated by the gyroscope is more reliable. On the contrary, when the linear motion acceleration is small, ${\dot{q}}_{gyr}$ decreased and *e*1 increased. Therefore, $\mu_{t}$ should be tuned to be larger.

The rules of the fuzzy inference are set according to the aforementioned principles in Table 1.

(3) Determine the weights and rule reliability

In practice, it is important to establish the relationship between the weights of the fuzzy rules and the reliability of the fuzzy rules in the knowledge base. There is a reversible mapping between the weights and the corresponding fuzzy rule confidence vectors.

(4) Choose the appropriate relationship generation method and inference synthesis algorithm

**Figure 6:**
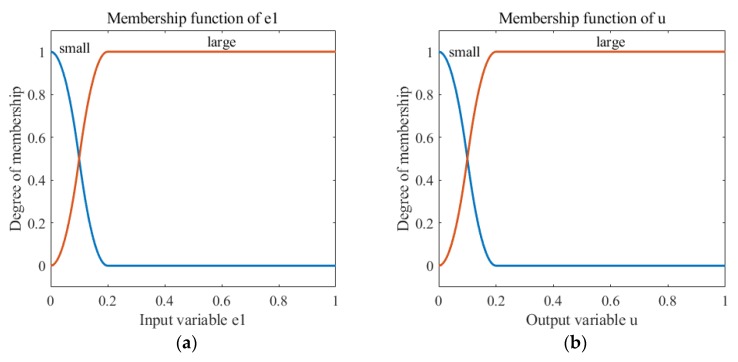
The fuzzy membership functions. (**a**) The input membership function. (**b**) The output membership function.

The selection of appropriate relationship generation methods and inference synthesis algorithms is required when designing a fuzzy controller. This article used the Z-shaped membership function and the S-shaped membership function in the MATLAB fuzzy toolbox. The fuzzy membership functions designed based on the fuzzy inference rules and rule reliability are shown in Figure 6.

(5) Defuzzification

When the output of an inference process forms a fuzzy output set, it is necessary to compress its distribution in order to produce a single value that expresses the output of the fuzzy system, that is, anti-blurring. This study used the maximum membership degree average method.

## 3. Experimental Results and Discussion

In this section, we consider the experimental testbed and evaluate the performance of the proposed FTECF method at different test times and motion speeds. We also compare the FTECF with two other methods in terms of accuracy and structure, so as to further evaluate its performance.

**Figure 7:**
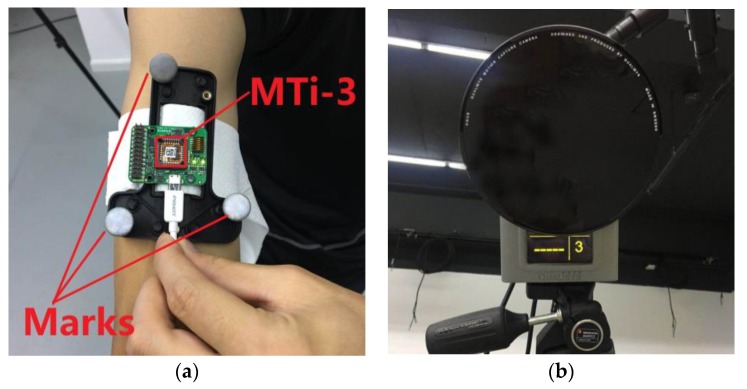
The experimental illustrations. (**a**) MTi-3 placed on the upper arm. (**b**) Oqus 7+.

To verify the proposed algorithm, we used the MARG sensors named MTi-3 [21]. MTi-3 is produced by Xsens Technologies (Enschede, The Netherlands). Figure 7a shows that we bound MTi-3 to the subject’s right upper arm to measure its motion. All of the sensor signals were sampled at 100 Hz and were interfaced to a personal computer (PC) via a universal serial bus (USB) cable. The accompanying software MT manager provided the calibrated sensor measurements. An Oqus 7+ optical motion capture system (Qualisys, Göteborg, Sweden), shown in Figure 7b, provided the reference orientation, which captures motion by using passive reflective technology with three cameras [22]. The spatial positioning accuracy of Oqus 7+ is less than 1 mm, and the latency time is less than 4 ms.

**Figure 8:**
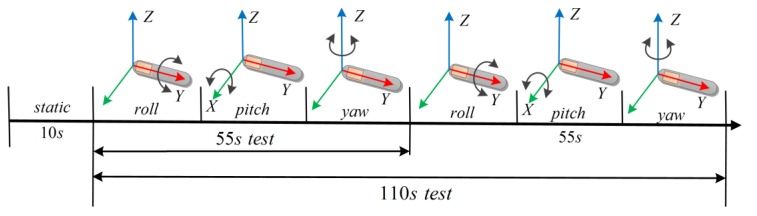
The experimental illustrations of motion.

In the experiment, the subject swung his upper arm according to the procedure of Figure 8. First, the subject stretched his upper arm and remained stationary at the T-pose for about 10 s, in order to calculate the initial position. Then, the subject swung his upper arm in the order of roll–pitch–yaw, and subsequently repeated the motion for about 55 s in the same order. As shown in Figure 8, we referred to the entire experiment as the 110 s test, and the approximately one-minute portion of the 110 s test as the 55 s test. By comparing the results of the two tests, we could conclude the influence of time on the algorithm. In addition, two motion speeds were mentioned in Section 2.3. In order to study the performance of the proposed algorithm at different motion speeds, we performed three trials for each speed.

Two other algorithms, Madgwick’s method [11] and Yun’s method [4], were used to compare the proposed FTECF method. In order to verify the orientation estimation accuracy, the root mean square error (RMSE) value of the Euler angles from the quaternion-based orientation was chosen as the criterion to evaluate the performance of the proposed FTECF method [4,5,6,10]. The calculation formula is as follows:

(22) $$e_{RMSE}=\sqrt{\frac{\sum_{i=1}^{n}{\left(\beta_{obs}-\beta_{tru}\right)}^{2}}{n}}$$

where $\beta_{obs}$ indicates the Euler angles calculated by the FTECF or other methods, the Euler angles, $\beta_{tru}$, are the reference attitudes, and *n* indicates the number of calculations.

Note that Yun’s method was assumed in the quasi-static state, which means that the acceleration of motion is small relative to the acceleration of gravity. The experiment obviously did not meet this assumption. We performed Equations (18) and (19) on the QUEST algorithm in Yun’s method (similar to the ESOQ-2 algorithm in this paper, which uses a gravitational acceleration vector and a geomagnetic vector to determine the attitudes).

The relevant parameter of the fuzzy tuned algorithm was selected as ξ=0.0006325. The corresponding thresholds for acceleration and angular velocity were $\delta_{a}=0.1\;{m/s}^{2}$ and $\delta_{w}=10{\;}^{{}^{\circ}}/s$, respectively. ‖g‖ and ‖h‖ were selected as $9.78\;{m/s}^{2}$ and 0.6 Gauss, respectively. The declination angle of the local geomagnetic vector ε was −58°.

### 3.1. The Effect of Motion Speed and Test Time on the Proposed FTECF

**Figure 9:**
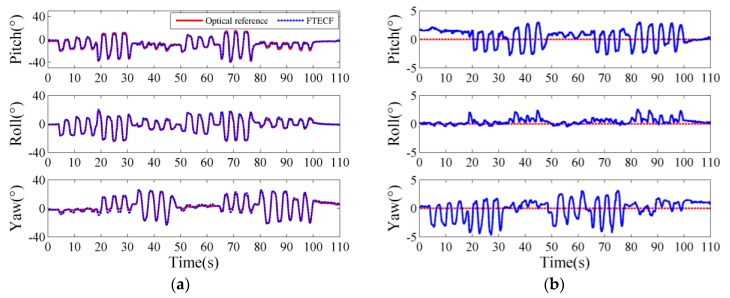
Typical results of the Fuzzy Tuned and Second EStimator of the Optimal Quaternion Complementary Filter (FTECF) at 0.5 mov/s. (**a**) The Euler angles of the optical and the proposed FTECF. (**b**) Euler angle errors.

**Figure 10:**
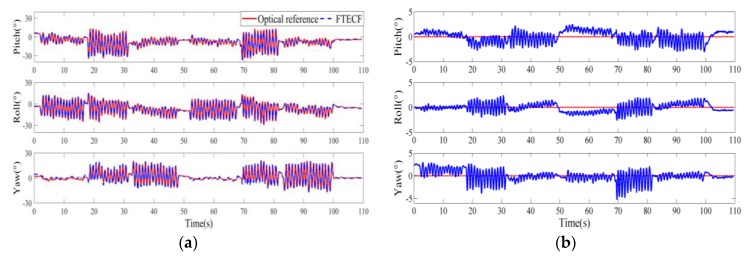
Typical results of FTECF at 2 mov/s. (**a**) The Euler angles of the optical and the proposed FTECF. (**b**) Euler angle errors.

We plotted the typical measurements of the proposed FTECF at two motion speeds. Figure 9 and Figure 10 are the typical measurement results of FTECF at 0.5 mov/s and 2 mov/s, respectively. In Figure 9a and Figure 10a, the red solid lines indicate the Euler angles measured by the optical motion capture system, and the blue dotted lines indicate the Euler angles calculated by the FTECF. The blue lines in Figure 9b and Figure 10b indicate the angle errors calculated by Equation (22). As can be seen from Figure 9b and Figure 10b, in terms of the fluctuation of the error, yaw was the largest, pitch was second, and roll was the smallest. In addition, the results showed that the proposed FTECF maintained a RMSE within a certain range of accuracy.

**Figure 11:**
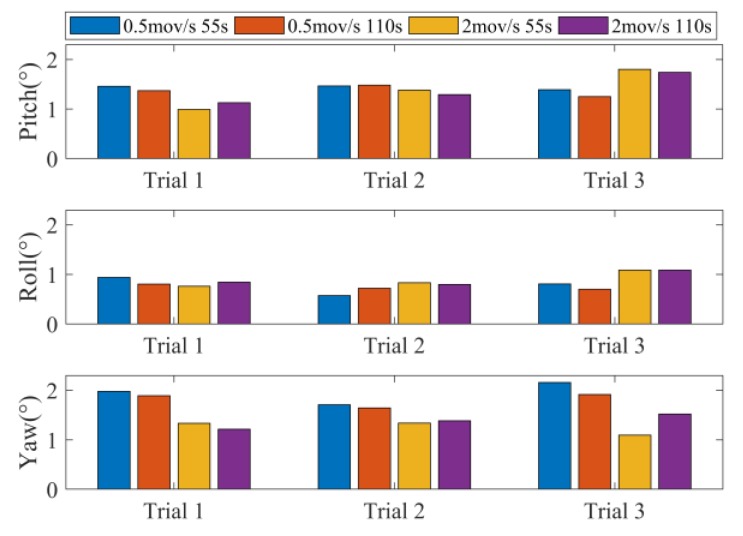
Root mean square error (RMSE) of the proposed FTECF method.

Figure 11 shows the RMSE of the FTECF under four different test conditions. In Figure 11, the blue bars indicate the RMSE of the FTECF at the 0.5 mov/s, 55 s test condition. The meaning of the red, yellow, and purple bars can be inferred from the legend. In order to obtain the influence of the motion speed on the proposed algorithm, we compared the blue and yellow bars with a test time of 55 s, and the red and purple bars with a test time of 110 s. The results show that yaw had a smaller RMSE at higher motion speeds, while pitch and roll changed less at different motion speeds when compared with yaw. Similarly, in terms of the influence of test time on the proposed algorithm, we compared the blue and red bars with a motion speed of 0.5 mov/s, and the yellow and purple bars with a motion speed of 2 mov/s. From Figure 11, we can see that the three Euler angles showed a small change at different test times.

### 3.2. Compare the Proposed FTECF and the Other Two Methods

To compare the performances of the proposed FTECF and the other two methods, we listed each of the Euler angle’s maximum RMSE from each method, as shown in Table 2. We also listed the maximum errors of each method. The maximum RMSE of each method and the smallest maximum error of the three methods are in bold.

Table 2 shows that the maximum RMSE of the FTECF was less than 2.2°, and the maximum error was less than 5.4°. In addition, the RMSE of the pitch and roll of the FTECF were also smaller than the other two methods, while the RMSE of the yaw was slightly larger than that in Madgwick’s method.

The proposed FTECF found a balance between high precision and simple structure. Compared with the Kalman filter algorithm represented by Yun’s method, the proposed method needed lower calculation costs and adapted well to high-speed human motion. The gradient descent algorithm represented by Madgwick’s method was also simple in structure. However, the proposed method had a higher precision than Madgwick’s method.

## 4. Conclusions

In this paper, we proposed a complementary filter, based on fuzzy logic and the ESOQ-2 algorithm, for human motion measurement. Firstly, the proposed method was effective at handling high dynamic movement with the fuzzy tuned algorithm and the compensation of the accelerator. The proposed algorithm did not accumulate errors under either 55 s and 110 s of measurement, indicating that it had the potential for long-term human motion measurement. Secondly, the RMSE of the proposed FTECF was less than 2.2°, which was comparable to the other two methods. In summary, this paper demonstrated the different speed motion measurements of the human upper arm by using the proposed method, and the results also illustrated its high accuracy.

Due to its high accuracy and computational efficiency, the proposed algorithm can be potentially implemented in a network of miniature MARG sensors for human body motion, forming a truly portable and ambulatory motion measurement system. The motion measurement system can be used in patient rehabilitation and behavioral monitoring. Future work will further study the influence of magnetic interference on the proposed algorithm. The complexity of the algorithm and the measurement effect for a longer time will also be studied. In addition, we will study the joint orientation assessment when the sensors are used in combination.

## Figures and Tables

**Table 1:** Fuzzy rules.

*e*1	u
small	small
large	large

The inference rule language is expressed as follows: Rule 1—if *e*1 is small, then u is small; Rule 2—if *e*1 is large, then u is large.

**Table 2:** Summary of errors. RMSE—root mean square error; FTECF—Fuzzy Tuned and Second EStimator of the Optimal Quaternion Complementary Filter.

Euler Angles (°)	FTECF	Madgwick’s Method	Yun’s Method
RMSE (pitch)	1.8024	**2.2313**	1.9024
RMSE (roll)	1.0884	1.2695	1.4793
RMSE (yaw)	**2.1605**	2.1393	**2.5881**
Maximum error	**5.376**	5.801	9.463
